# Ethnic differences in protein biomarkers of peripartum cardiomyopathy: a proteomic study on the EORP cohort

**DOI:** 10.1002/ehf2.15419

**Published:** 2025-09-04

**Authors:** Vitaris Kodogo, Karen Sliwa, Alice M. Jackson, Hasan Al‐Farhan, Sorel Goland, Jasper Tromp, Peter van der Meer, Kamilu Karaye, Alexandre Mebazaa, Johann Bauersachs, Liam Bell, Julian Hoevelmann, Charle Viljoen, the EurObservational Research Programme in conjunction with the Heart Failure Association of the European Society of Cardiology Study Group on Peripartum Cardiomyopathy

**Affiliations:** ^1^ Cape Heart Institute, Faculty of Health Sciences University of Cape Town Cape Town South Africa; ^2^ Department of Internal Medicine University of Zimbabwe Harare Zimbabwe; ^3^ Department of Medicine, Division of Cardiology, Faculty of Health Sciences, Groote Schuur Hospital University of Cape Town Cape Town South Africa; ^4^ School of Cardiovascular and Metabolic Health University of Glasgow Glasgow UK; ^5^ University of Baghdad College of Medicine, Iraqi Board for Medical Specializations, Baghdad Heart Centre Baghdad Iraq; ^6^ Heart Institute, Kaplan Medical Center, Rehovot, affiliated to the Hebrew University Jerusalem Israel; ^7^ Saw Swee Hock School of Public Health & the National University Health System Singapore Singapore; ^8^ Duke‐NUS Medical School Singapore Singapore; ^9^ Department of Cardiology, University Medical Center Groningen University of Groningen Groningen The Netherlands; ^10^ Department of Medicine Bayero University Kano Nigeria; ^11^ APHP, Department of Anesthesiology and Critical Care Saint Louis Lariboisière Hospitals Paris France; ^12^ Department of Cardiology and Angiology Hannover Medical School Hannover Germany; ^13^ Centre for Proteomic and Genomic Research Cape Town South Africa; ^14^ Department of Internal Medicine III ‐ Cardiology, Angiology and Intensive Care Medicine Saarland University Hospital, Saarland University Homburg Germany

**Keywords:** Biomarkers, Ethnicity, Proteomics, PPCM

## Abstract

**Aims:**

The diagnosis of peripartum cardiomyopathy (PPCM) is often delayed due to the absence of disease‐specific biomarkers. Recently, serum proteins—QSOX1, adiponectin (ADIPOQ) and ITIH3—have shown potential for improving diagnostic accuracy, especially when used with NT‐proBNP. However, the influence of ethnicity on their expression remains unclear. We aimed to assess whether serum biomarker profiles differ among ethnic groups in a multinational PPCM cohort.

**Methods and results:**

Eighty‐two PPCM patients from seven countries in the EURObservational Research Programme (EORP) provided demographic data and serum samples. Ethnicity was self‐reported. Proteomic profiling at diagnosis was performed using DIA‐based label‐free LC–MS, and data were analysed with Spectronaut v15. Ethnic variation was evaluated through principal component analysis (PCA). Participants had a mean age of 30.5 ± 6.7 years; 75% had no hypertension during pregnancy. Median LVEF was 35% (IQR 27.0–41.1), with no ethnic differences. Middle Eastern women showed more severe LV dilatation. PCA revealed no significant clustering by ethnicity; PC1 and PC2 explained 15.2% and 12.0% of variance, respectively.

**Conclusions:**

QSOX1, ADIPOQ and ITIH3 exhibited consistent expressions across ethnic groups, supporting their use as universal PPCM biomarkers.

## Introduction

Peripartum cardiomyopathy (PPCM) is a form of heart failure occurring late in pregnancy or shortly after delivery, without other identifiable causes.^1^ A multihit model suggests that susceptible women—due to genetic predisposition, extreme maternal age or multifetal gestation—experience oxidative stress, leading to disrupted angiogenic and metabolic pathways and ultimately heart failure.^2^, ^3^ PPCM diagnosis is often delayed because its symptoms, like dyspnoea and oedema, mimic normal pregnancy discomforts. The absence of disease‐specific biomarkers compounds this delay.^4^ Timely diagnosis is essential because targeted therapies are available.

Recent proteomic studies identified several promising biomarkers for PPCM.^5^ Notably, ADIPOQ, QSOX1 and ITIH3, identified in the EURObservational Research Programme Registry (EORP), show high diagnostic accuracy, particularly when used in combination with NT‐proBNP.^6^ However, cardiovascular protein biomarkers often exhibit ethnic variation. This study investigated whether ethnic differences influence levels of recently identified PPCM biomarkers.^7^, ^8^, ^9^

## Material and methods

### Study sample

This study conformed with the Declaration of Helsinki, and ethical approval was obtained from the University of Cape Town's Human Research Ethics Committee (R033/2013).

The patients were enrolled in the EORP registry for PPCM during the baseline visit at participating centres across seven different nations.^10^ As part of the diagnostic work‐up, every participant had a transthoracic echocardiography (TTE) confirming a left ventricular ejection fraction of less than 45% (LVEF <45%) and a confirmed PPCM diagnosis. All the PPCM patients presented postpartum and were recruited within 6 months after delivery. The demographic and clinical data and serum samples for this study were collected from all patients before initiating any heart failure‐related medication. Ethnicities were self‐declared and were grouped into Caucasian, African, Middle Eastern and mixed ancestry.

Serum samples were frozen immediately and were stored at −80°C without thawing until protein analysis. The samples were shipped on dry ice from the different centres to the Cape Heart Institute in South Africa.

### Proteomic profiling of PPCM patients

The methods for serum proteomics profiling, proteomics analysis with liquid chromatography–tandem mass spectrometry, and sample preparation for library generation used in this study have been described previously.^6^ In summary, serum samples were processed, digested and analysed at the Centre for Proteomic and Genomic Research in South Africa. Proteome profiling was done using data‐independent acquisition‐based label‐free quantitative liquid chromatography. A study‐specific SWATH (sequential window acquisition of all theoretical fragment ion spectra) library was generated from a pooled sample made from an aliquot of each sample. All samples underwent depletion before analysis.

### Statistical analysis

Where appropriate, clinical data for continuous variables were expressed as mean ± standard deviation (SD) or median and interquartile range (IQR). Ethnic groups were compared using one‐way ANOVA. Categorical clinical data were compared between the four ethnic groups using the chi‐square or Fisher exact tests (when cell values were <5 or where the column marginal values were uneven). Normalized protein intensities were compared between PPCM patients and healthy controls to identify significantly changed proteins according to our previous findings.^6^ Feature selection and classification of the changed proteins were performed by Boruta R packages (R Foundation). Boruta performed 499 iterations (maxRuns = 500), which identified seven proteins that are considered important in PPCM diagnosis.^6^ However, sensitivity and specificity tests narrowed down to ADIPOQ, QSOX1 and ITIH3 as potential diagnostic markers of PPCM.

### Principal component analysis (PCA)

In this study, PCA was utilized to analyse how the protein abundances of ADIPOQ, QSOX1 and ITIH3—identified as potential diagnostic markers in our previous research—deviate from the mean across the four ethnic groups. Based on the relationship (covariances) between the ethnic groups and the covariance matrix, the two most significant possible variances in the data sets (principal components 1 and 2 [PC1 and PC2]) were determined.

## Results

### Clinical characteristics of PPCM patients of different ethnic groups

In this study, 82 patients with PPCM were recruited across four ethnic groups [African (47.6%), Caucasians (19.5%), Middle Eastern (13.4%) and mixed ancestry (19.5%)]. The clinical characteristics of the patients with PPCM are summarized in *Table* *1*. In brief: The African cohort was significantly younger (27.8 ± 6.4 years) compared to the other ethnic groups (Caucasians: 34.1 ± 5.9 years, Middle Eastern: 33.2 ± 7.2 years, mixed ancestry: 31.6 ± 5.3 years, *P* = 0.003). In terms of New York Heart Association (NYHA) functional class, 52.4% of the total cohort was in class III/IV. The Caucasians were most likely to present in NYHA class III/IV (75.0%) (*P* = 0.05). However, the Middle Eastern group had the highest BMI (31.2 ± 5.2 kg/m^2^), compared with the African (25.1 ± 8.3 kg/m^2^), Caucasian (23.9 ± 4.1 kg/m^2^), and mixed ancestry (24.3 ± 5.0 kg/m^2^) groups (*P* = 0.037). Although there were no significant differences in blood pressure, the Middle Eastern group had the highest heart rate (114.0 b.p.m.), which was significantly higher than the African (103.5 b.p.m.), Caucasian (80.0 b.p.m.) and mixed ancestry (82.5 b.p.m.) groups (*P* = 0.023). The Middle Eastern group also had the largest left ventricular end‐diastolic diameter (LVEDD) at 64.0 mm, compared to 58.0 mm in the African group, 52.5 mm in Caucasians, and 57.0 mm in the mixed ancestry group (*P* = 0.006). No significant differences in LVEF were observed across the groups.

**Table 1:** Clinical variables of patients with PPCM from different ethnic groups

	Total	African	Caucasian	Middle Eastern	Mixed ancestry	
	*N* = 82	*N* = 39	*N* = 16	*N* = 11	*N* = 16	*P*‐value
Age (years)	30.5 ± 6.7	27.8 ± 6.4	34.1 ± 5.9	33.2 ± 7.2	31.6 ± 5.3	0.003
Parity						0.39
1	55 (76.4)	25 (69.4)	9 (75.0)	9 (81.8)	12 (92.3)	
≥2	17 (23.6)	11 (30.6)	3 (25.0)	2 (18.2)	1 (7.7)	
Hypertension during pregnancy						0.67
No hypertension	61 (74.4)	27 (69.2)	12 (75.0)	8 (72.7)	14 (87.5)	
Hypertension without pre‐eclampsia	12 (14.6)	8 (20.5)	2 (12.5)	2 (18.2)	0 (0.0)	
Pre‐eclampsia	9 (11.0)	4 (10.3)	2 (12.5)	1 (9.1)	2 (12.5)	
Prior PPCM	9 (13.8)	4 (14.3)	1 (7.7)	1 (11.1)	3 (20.0)	0.81
BMI (kg/m^2^)	25.6 ± 7.0	25.1 ± 8.3	23.9 ± 4.1	31.2 ± 5.2	24.3 ± 5.0	0.037
NYHA functional class						0.05
I/II	39 (47.6)	20 (51.3)	4 (25.0)	5 (45.5)	10 (63.5)	
III/IV	43 (52.4)	19 (48.7)	12 (75.0)	6 (54.5)	6 (37.5)	
Systolic BP (mmHg)	115 (100–130)	118 (100–130)	113.5 (107–123)	100.0 (98–120)	127.5 (109–140)	0.20
Diastolic BP (mmHg)	79.0 (70–88)	80.0 (70–90)	71 (65–80)	73.5 (69.0–86.0)	80.0 (70.0–88.5)	0.52
Heart rate (b.p.m.)	96 (80–115)	103.5 (90–114)	80.0 (78–115)	114.0 (81–129.0)	82.5 (64.0–99.0)	0.023
QTc by Bazett (ms)	454.9 (421.1–473.0)	458.2 (439.7–470.4)	468.2 (440.4–481.1)	416.9 (391.0–447.1)	455.8 (416.0–479.6)	0.089
LVEDD (mm)	58.0 (52.0–64.0)	58.0 (54.0–66.0)	52.5 (48.0–57.5)	64.0 (62.0–65.0)	57.0 (49.0–63.5)	0.006
LVESD (mm)	49.0 (43.0–55.0)	49.0 (43.0–55.0)	42.5 (34.0–47.0)	53.0 (49.0–56.0)	46.0 (42.5–55.0)	0.098
LVEF (%)	35.0 (27.0–41.1)	36.0 (27.0–45.0)	36.0 (25.0–40.5)	34.0 (28.0–38.0)	32.5 (27.0–37.5)	0.68
Relative abundance						
ADIPOQ	3.23(0.15–6.79)	3.57(0.87–6.79)	3.16(0.15–5.86)	3.18(1.68–4.77)	2.64(0.50–4.06)	0.09
QSOX1	3.54(2.55–5.19)	3.71(2.60–5.19)	3.5 (2.55–4.68)	3.36(2.96–3.91)	3.38(2.58–4.19)	0.103
ITIH3	4.53(3.62–5.72)	4.52 (3.62–5.72)	4.59(3.88–5.36)	4.58(3.88–5.35)	4.48 (4.05–5.38)	0.886

Values are mean ± SD or median and IQR or *n* (%).

BMI, body mass index; BP, blood pressure; LVEDD, left ventricular end‐diastolic diameter; LVEF, left ventricular ejection fraction; LVESD, left ventricular end‐systolic diameter; NYHA, New York Heart Association Class; QTc, QT corrected for heart rate; PPCM, peripartum cardiomyopathy.

### Principal component analysis

**Figure 1:**
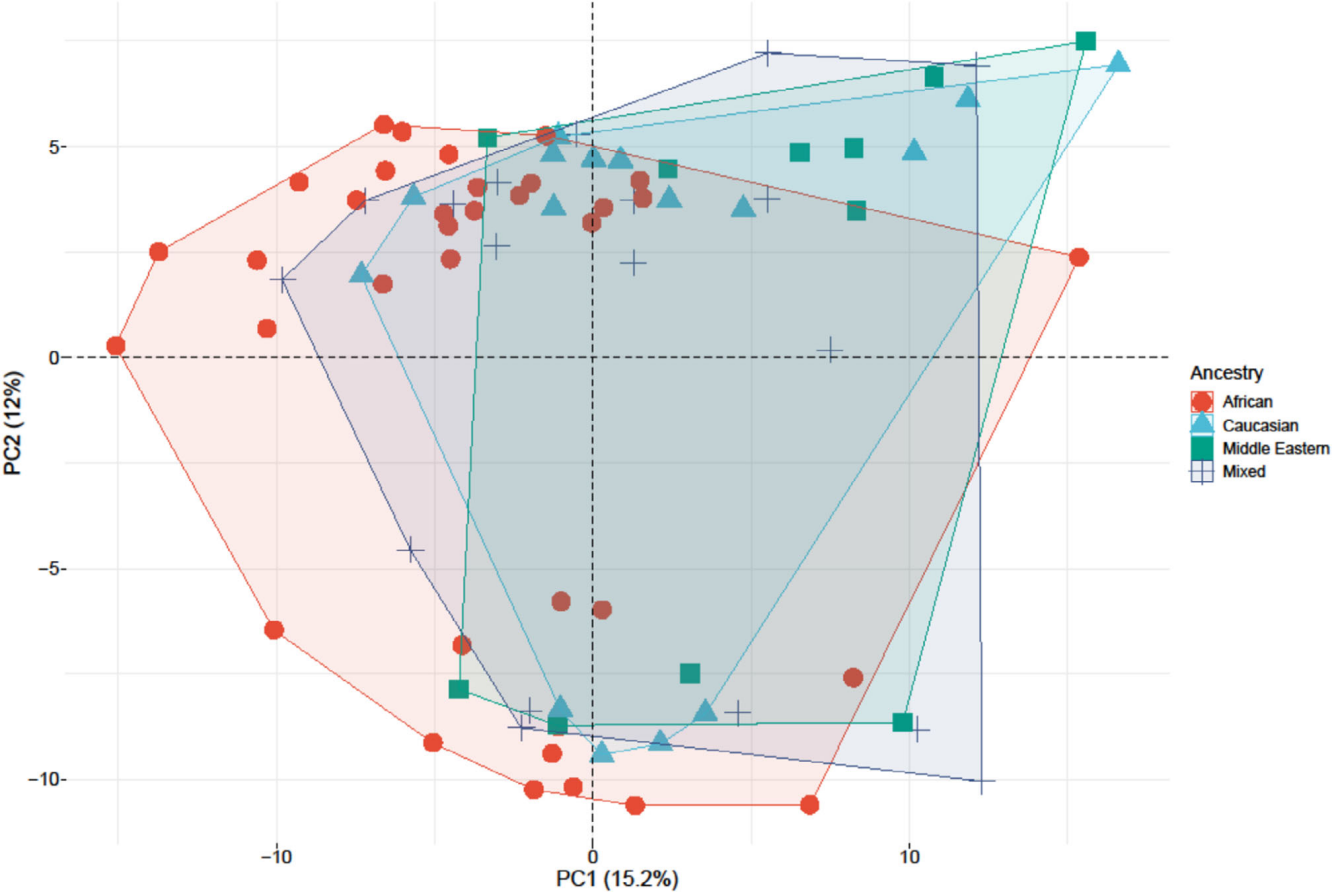
Principal component analysis (PCA) of the proteomic biomarkers of PPCM patients from different ethnic groups. Factor map of the PCA performed on 82 PPCM patient samples and 3 protein variables. No separate cluster groups were identified corresponding to African (red), Caucasian (blue), Middle Eastern (green) or mixed ancestry (grey). The first two components explained 15.2% and 12.0% of the variances, respectively.

PCA is a statistical method utilized for reducing dimensionality while maintaining the maximum variance in the data. In this study, PCA combined with unsupervised hierarchical clustering indicated that the variability in protein expression patterns was minimal among different ethnic groups (see *Figure* *1*). Specifically, the first principal component (PC1) explained only 15.2% of the total variability across ethnic groups, while the second principal component (PC2) contributed an additional 12.0%.

## Discussion

This multicenter proteomics study assessed ethnic differences in the expression of newly identified PPCM biomarkers ADIPOQ, QSOX1 and ITIH3. No significant ethnic differences were observed, suggesting these biomarkers are applicable across diverse populations.

The incidence of PPCM varies significantly across different countries and regions.^11^ Ethnicity may be a contributing factor to these differences. Previous studies indicate that African and Native American women have a higher risk of developing PPCM compared to Caucasian women, even after adjusting for socioeconomic factors.^12^ Additionally, African patients tend to have a worse prognosis, characterized by lower rates of left ventricular recovery, higher resource utilization and increased mortality.^13^, ^14^ Clinically, African patients are typically younger and more likely to present with an LVEF of less than 30%. This may be attributed to a younger African obstetric population that seeks medical care later, allowing the disease to progress before receiving appropriate treatment.

Nevertheless, it is striking that PPCM has similar clinical features worldwide, even if it is an epidemiologically heterogeneous illness.^15^ Most studies which compared African and Caucasian PPCM patients report comparable baseline characteristics.^12^, ^16^ Furthermore, recent findings from the EORP PPCM registry suggest that the demographics of patients with PPCM do not differ in different socioeconomic groups.^17^ However, Sliwa et al. suggest that outcomes for mothers and newborns are influenced by socioeconomic factors specific to each country.^17^ High mortality rates in low‐ and middle‐income countries may be attributed to limited resources and delayed presentation. However, if patients are diagnosed within 10 days of symptom onset, up to 65% will experience recovery of left ventricular function.^18^ Identifying new diagnostic biomarkers will facilitate early detection, which in turn could improve prognosis and enhance disease outcomes.

ADIPOQ is a 244‐amino‐acid adipokine with a molecular weight of approximately 26 kDa, playing a critical role in energy metabolism, insulin sensitivity and inflammation. During the progression of heart failure, ADIPOQ levels increase with disease severity, with elevated levels being associated with poor prognosis. Consistent with our findings, ADIPOQ levels measured in African American and Caucasian women across different body mass index (BMI) categories were comparable among Caucasian obese women, African obese women and African American non‐obese women.^19^ However, in the same study, ADIPOQ levels were found to be significantly higher in Caucasians and non‐obese women. Similarly, the Manhattan Study reported lower ADIPOQ levels among Black and Hispanic individuals and those with various vascular risk factors, while levels were higher in older individuals.^20^

No study has directly compared QSOX1 and ITIH3 levels in different ethnic groups. Nonetheless, QSOX1 was shown to be upregulated in the heart upon acute heart failure (AHF).^21^, ^22^ Further studies are required to elucidate the pathophysiological roles of ITIH3 in cardiovascular disease and to support its potential use as a biomarker.

This study's strength lies in its multiethnic cohort, enhancing the generalizability of the results. With validation in larger populations, proteomic profiling could support the development of diagnostic tools (e.g., test strips) enabling early PPCM detection. This would facilitate earlier treatment, including bromocriptine,^23^ and guideline‐directed heart failure therapy—and improve outcomes.

## Conclusions

ADIPOQ, QSOX1 and ITIH3 significantly enhance the diagnostic accuracy of PPCM. This study found no ethnic variation in their expression, supporting their use in diverse patient populations. These biomarkers could aid early PPCM diagnosis and improve maternal health outcomes.

## Funding

Open Access funding enabled and organized by Projekt DEAL.
